# *Batrachochytrium salamandrivorans* Threat to the Iberian Urodele Hotspot

**DOI:** 10.3390/jof7080644

**Published:** 2021-08-07

**Authors:** Jaime Bosch, An Martel, Jarrod Sopniewski, Barbora Thumsová, Cesar Ayres, Ben C. Scheele, Guillermo Velo-Antón, Frank Pasmans

**Affiliations:** 1 Biodiversity Research Institute (IMIB), University of Oviedo-Principality of Asturias-CSIC, 33600 Mieres, Spain; barbora.thums@gmail.com; 2 Museo Nacional de Ciencias Naturales-CSIC, 28006 Madrid, Spain; 3 Wildlife Health Ghent, Department of Pathology, Bacteriology and Poultry Diseases, Ghent University, B9820 Merelbeke, Belgium; an.martel@ugent.be (A.M.); frank.pasmans@ugent.be (F.P.); 4 Fenner School of Environment and Society, Australian National University, Canberra 2601, Australia; u7093545@anu.edu.au (J.S.); ben.scheele@anu.edu.au (B.C.S.); 5 Asociación Herpetologica Española, 28006 Madrid, Spain; cesar@herpetologica.org; 6 CIBIO/InBIO, Centro de Investigação em Biodiversidade e Recursos Genéticos, Universidade do Porto, 4485-661 Vairão, Portugal; guillermo.velo@gmail.com; 7 Grupo GEA, Departamento de Ecoloxía e Bioloxía Animal, Universidade de Vigo, 36310 Vigo, Spain

**Keywords:** chytridiomycosis, amphibian chytrid fungus, salamanders, Spain, Portugal, threat, conservation-units, surveillance, biosecurity

## Abstract

The recent introduction of the chytrid fungus *Batrachochytrium salamandrivorans* into northeastern Spain threatens salamander diversity on the Iberian Peninsula. We assessed the current epidemiological situation with extensive field sampling of urodele populations. We then sought to delineate priority regions and identify conservation units for the Iberian Peninsula by estimating the susceptibility of Iberian urodeles using laboratory experiments, evidence from mortality events in nature and captivity and inference from phylogeny. None of the 1395 field samples, collected between 2015 and 2021 were positive for *Bsal* and no *Bsal*-associated mortality events were recorded, in contrast to the confirmed occurrence of *Bsal* outbreak previously described in 2018. We classified five of eleven Iberian urodele species as highly susceptible, predicting elevated mortality and population declines following potential *Bsal* emergence in the wild, five species as intermediately susceptible with variable disease outcomes and one species as resistant to disease and mortality. We identified the six conservation units (i.e., species or lineages within species) at highest risk and propose priority areas for active disease surveillance and field biosecurity measures. The magnitude of the disease threat identified here emphasizes the need for region-tailored disease abatement plans that couple active disease surveillance to rapid and drastic actions.

## 1. Introduction

Emerging fungal wildlife diseases are increasingly threatening biodiversity [1,2]. A prominent example is the amphibian disease chytridiomycosis, which has contributed to numerous amphibian declines in the Americas and Australia, and some declines in Europe [3,4,5,6]. Chytridiomycosis is caused by the chytrid fungi *Batrachochytrium salamandrivorans* (*Bsal*) and *B. dendrobatidis* (*Bd*), which are both thought to have originated in East Asia and to have been spread by humans to Europe [7,8]. Since the description of *Bsal* in 2013 in the Netherlands, it has been detected in adjacent areas of Belgium, France and Germany [9,10]. The severe decline of the fire salamander (*Salamandra salamandra*) from infected regions has generated elevated concern for the persistence of other salamander species in Europe as *Bsal* continues to spread [11].

In 2018, a disjunct *Bsal* outbreak was detected in the Iberian Peninsula in northeastern Spain, nearby to Europe’s most threatened newt species, the Montseny brook newt (*Calotriton arnoldi*) [12]. Two other recent reports have reported positive qPCR results in north central Spain [13,14]. With seven genera, ten species and a high number of small-range endemic lineages, the Iberian Peninsula is a hotspot of diversification of the family Salamandridae. As such, the recent incursion of *Bsal* into the region may pose an acute threat to this peninsula’s urodele diversity. Mitigating potential impacts requires a better understanding of *Bsal*’s distribution in the Iberian Peninsula and species susceptibility.

The impact of *Bsal* introduction is determined by a complex interplay between host and pathogen in a given environmental context [15,16,17,18,19]. *Bsal* infection results in a wide spectrum of outcomes, with consistent, low-level and asymptomatic infections in some species, variable levels and disease courses in others and consistently lethal infections in the most vulnerable, hypersusceptible species [11]. While the mechanisms underpinning infection and disease dynamics are incompletely understood, several (potential) drivers have been identified. For example, host susceptibility to lethal disease varies with life stage, species, infectious dose, thermal ecology and prior infections [15,17,20]. Further, biotic and abiotic environmental pathogen reservoirs contribute to the rapid population decline observed in hypersusceptible species [21].

The successful mitigation of *Bsal* impacts in the Iberian Peninsula requires early detection of disease, coupled with fast and sustained implementation of drastic control measures to contain the pathogen. Mitigation actions can be informed by threat analyses, which can guide preventive and remedial conservation efforts, with the ultimate goal of ensuring the long-term persistence of the unique Iberian urodele diversity. Threat assessment requires awareness of the factors governing the probability of pathogen introduction and expected impact. Potential avenues for *Bsal* introduction include infected amphibian hosts (either from neighboring and infected populations or after release by humans), nonamphibian hosts (e.g., waterfowl) and fomites through passive transport. The trade in live amphibians has probably vectored *Bsal* into Europe from its origin in Asia [11,22,23] and is likely to have contributed to long-distance spread across Europe and introduction in Spain [12]. The latter may explain the current, rather erratic distribution pattern of *Bsal* in Europe and suggests the potential of novel introductions and disease outbreaks throughout *Bsal’s* predicted niche [17,24].

Here, we estimate the risk *Bsal* poses to Iberian urodeles by (i) assessing the current distribution of the pathogen and epidemiological situation, (ii) estimating the susceptibility of Iberian urodeles using laboratory experiments and (iii) inferring susceptibility based on phylogenetic relationships. We then combine this information with overlays of species distributions to delineate priority regions and identify conservation units for the Iberian Peninsula.

## 2. Materials and Methods

### 2.1. Distribution of Bsal in the Iberian Peninsula

Between 2015–2021, we opportunistically sampled 66 populations of 10 Iberian urodele species, with a total of 1395 individuals sampled (Table S1). Recent reports of *Bsal*-positive qPCRs from Asturias [13,14] resulted in more intensive sampling efforts in this region, where we resampled two localities previously reported as positive. The vast majority of samples derived from surveys of live urodeles, with 21 samples from dead animals. Animals were sampled using cotton-tipped swabs and the swabs were examined for the presence of *Bsal* DNA using qPCR as described elsewhere [25]. In some cases, toe clips were collected and processed for qPCR. DNA was extracted from tissue using the DNAeasy Blood and Tissue kit (Qiagen GmbH, Hilden, Germany) following the manufacturer’s protocol. All animals were released on site after sampling.

Samples were also collected following reports to the hotline in Spain and Portugal, through which amphibian mortality events can be reported. The reporting of amphibian mortality events to this hotline results in diagnostic testing for *Bsal*. The diagnosis of *Bsal* chytridiomycosis combines a positive qPCR results with histopathology of dead animals [26].

### 2.2. Infection Dynamics of Bsal in Iberian Urodeles

Infection dynamics and disease course were experimentally assessed in *Chioglossa lusitanica*, *Calotriton asper* and *Lissotriton boscai* using previously described methods [11]. We compared infection dynamics of *Bsal* between captive *Chioglossa lusitanica, Lissotriton boscai* and *Calotriton asper* (permits Xunta de Galicia, ref 6370/RX763724_24/0/18 and INAGA, ref 500201/24/2018/3311) with those from the reference urodele species for susceptibility: the fire salamander (*Salamandra salamandra*). For all species, five or six adult specimens were inoculated with 10^3^ zoospores of the *Bsal* type strain AMFP13/1 using an established protocol [11]. In addition, given recent positive qPCR results from the Palmate newt (*Lissotriton helveticus*) in northern Spain [13,14], we also infected 10 captive bred palmate newts with two additional *Bsal* isolates (isolates AMFP14/1 and AMFP15/1; 5 animals per isolate) to study infection dynamics in this species. An earlier infection trial has indicated that *L. helveticus* has low susceptibly to *Bsal* infection [11]. The experiment was approved by the ethics committee of the Faculty of Veterinary Medicine (Ghent University, Merelbeke, Belgium, 2013/10; 2013/79; 2017/75).

Briefly, animals were individually exposed for 24 h at 15 °C to 10^3^ zoospores of *Bsal* in 1 mL of water. Afterwards, the salamanders were housed individually in plastic containers lined with moist tissue and containing a PVC tube as a hiding place or, for *C. asper*, in plastic containers holding 2 cm of water and a PVC tube. Temperatures were kept constant at 15 °C and dim, natural light was provided. The terrestrial salamanders were fed two times weekly ad libitum with crickets (*Acheta domestica*) and the aquatic ones with *Tubifex* (*C. asper*). Infection loads were followed with weekly sampling of all animals using cotton-tipped swabs and subsequent qPCR analysis to quantify *Bsal* loads [25]. For *C. asper*, none of the animals were infected after the first exposure. Five of these animals were re-exposed one year later following the same protocol.

Susceptibility was considered “high” in cases of high infection loads, with consistent and elevated mortality (>80%) in lab trials. “Intermediate” susceptibility was attributed to those taxa that demonstrated low or variable mortality in lab trials. Susceptibility was considered low in cases of low-level infections and the absence of overt clinical signs and mortality. Where data of disease outbreaks in captivity and/or nature were available, these were added for a more comprehensive assessment of the level of susceptibility. Where available, we added data of susceptibility for known invasive urodeles in the Iberian Peninsula: *Triturus anatolicus* and *Ommatotriton nesterovi/ophryticus*, which could potentially play a role in disease ecology.

### 2.3. Phylogenetic Influence upon Urodele Susceptibility to Bsal

We used the *phytools* v0.7.80 *packag*e [27] to evaluate the potential phylogenetic signal of urodele susceptibility to *Bsal* infection, based on susceptibility as outlined above for all European urodeles. We first converted lab-determined susceptibility to an ordinal scale, with “low” susceptibility recorded as 1, “intermediate” as 2 and “high” as 3, before using the *phylosig* function to calculate phylogenetic signal. The Jetz and Pyron phylogeny [28] was used, and the process repeated 1000 times, each time employing a randomly selected tree from the phylogeny.

### 2.4. Defining Priority Areas and Conservation Units for Iberian Urodeles

For all Iberian species, previously published data were included from infection trials and from disease outbreaks in nature or captivity and combined with the infection trials and the phylogenetic modeling described above to classify susceptibility (Table S2). Three lines of direct evidence (lab trials, mortality events in the field and mortality events in captivity) were available for three species (*Ichthyosaura alpestris, Triturus marmoratus* and *Salamandra salamandra*). *Bsal*-associated mortality was observed in *Lissotriton boscai* in a lab trial and during an outbreak in captivity. A single line of evidence of susceptibility derived from lab trials was available for five species *(Calotriton arnoldi, C. asper, Chioglossa lusitanica, Lissotriton helveticus, Pleurodeles waltl*) and susceptibility in *Triturus pygmaeus* was estimated based on a mortality event in captivity only. No information was available for *Lissotriton maltzani*.

For all species, we defined or estimated the range of conservation units to identify small-range susceptible lineages at the highest risk of extirpation in case of *Bsal* invasion (Table 1). Delineation of conservation units was based on defining evolutionary significant units (ESUs). ESUs were here defined as distinct Pleistocene lineages, which were retrieved from published phylogenetic/phylogeographic studies of Iberian urodeles (see Table S1). Diversification of Iberian urodeles (as for most of ectothermic Iberian taxa) mostly occurred during the Pleistocene climatic oscillations that led to population fragmentation and species survival within climatically stable areas (i.e., refugia) across the heterogeneous Iberian topography, followed by genetic differentiation and the formation of parapatric and allopatric distributions within species. While deeper (Pliocene) and more recent events (Holocene and Anthropocene) of diversification also shape the current genetic diversity and structure of Iberian urodeles, the Pleistocene is the dominant force, and the most studied geological epoch for salamander diversification in Iberia.

Small range was defined as occupancy of 100 or less 10 km × 10 km, using published databases (https://siare.herpetologica.es/bdh/distribucion for Spain and http://www2.icnf.pt/portal/pn/biodiversidade/patrinatur/atlas-anfi-rept for Portugal), both accessed 6 August 2021). We then compiled a species richness map of those Iberian urodeles considered susceptible to *Bsal* to identify regions with the estimated highest impact of *Bsal* on biodiversity and areas with high-risk conservation units. Using the *raster* v3.4.13 [29], *rgdal* v1.5.23 [30] and *sf* v1.0.0 [31] packages, we first defined the ranges of Iberian urodeles using raster files (10 × 10 km) obtained from the above national databases. To each raster we gave a normalized value of risk related to *Bsal* susceptibility as outlined in Table S2. Each raster was overlaid, and the species richness of each 2.5 arcminute grid cell also normalized. For each cell, the mean was calculated between the normalized species richness and normalized risk, such that cells with higher values are deemed to have higher conservation value.

## 3. Results

### 3.1. Bsal Is Currently Confirmed from a Single Outbreak Site on the Iberian Peninsula

**Figure 1:**
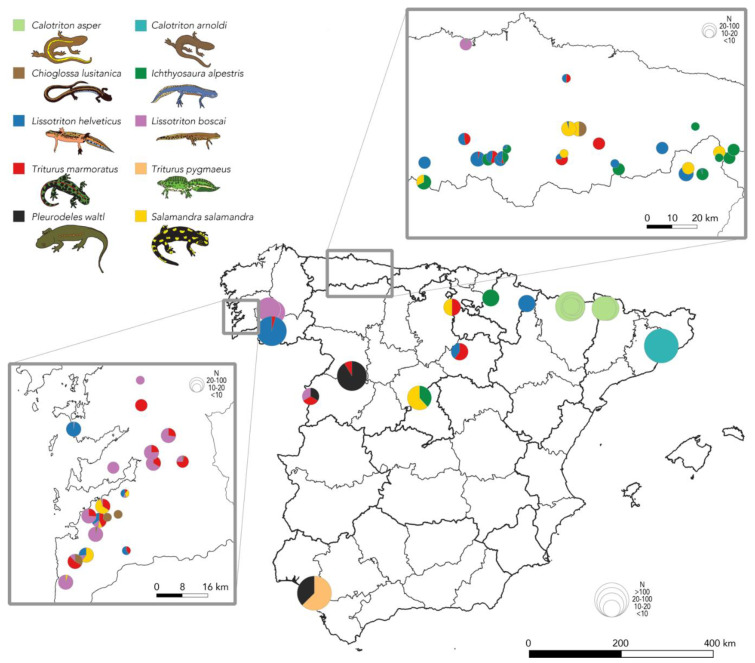
Map of Spain showing the location of urodele populations sampled between 2015 and 2021 for the presence of *Bsal* infection. None of the 1395 samples collected were positive. An intensive sampling effort was conducted in northern Spain due to recent reports of *Bsal* positive qPCRs from that area. Pie charts represent the proportion of sampled individuals of each species per location, and the sample size at each locality is indicated by the size of the pie chart.

None of the 1395 samples, collected between 2015 and 2021, were positive for *Bsal* (Figure 1). An intensive sampling effort was conducted in northern Spain (Asturias and southwestern Galicia) due to recent reports of *Bsal-*positive qPCRs from Asturias and its neighbor Cantabria [13,14]. However, *Bsal* was not detected in any of the 326 samples collected in this region. Between November 2020 and June 2021, seven cases of amphibian mortality events affecting three species (*Salamandra salamandra*, *Triturus marmoratus* and *Lissotriton helveticus*), were reported from the Cantabrian range to the hotline and examined for the presence of *Bsal*. None yielded the detection of *Bsal*.

### 3.2. Infection and Disease Dynamics of Bsal in Iberian Urodeles

**Figure 2:**
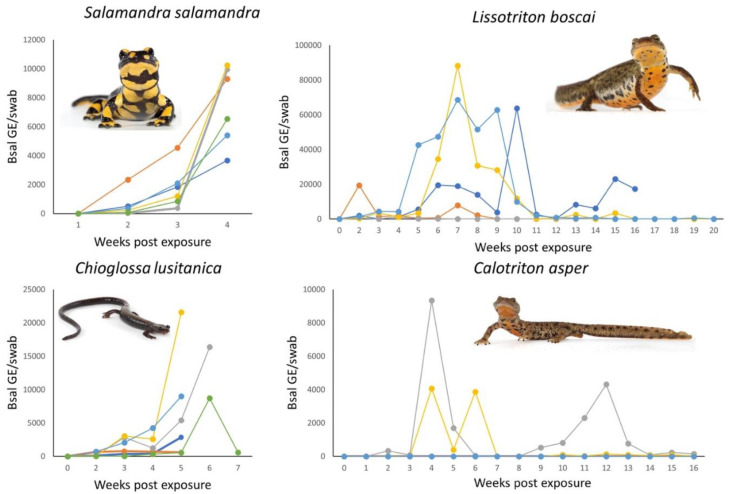
Overview of infection dynamics of *Bsal* after experimental exposure of salamanders to a single dose of 10^3^ zoospores. Each line represents the infection dynamics of a single animal. All *S. salamandra* and *C. lusitanica* died, compared to one *L. boscai* and no *C. asper*. Infection loads are expressed as genomic equivalents (GE) of *Bsal* per swab per timepoint.

Exposure of six *S. salamandra* to the *Bsal* type strain resulted in buildup of lethal *Bsal* infections (experiment ended at four weeks post inoculation to comply with humane endpoints). All six *C. lusitanica* died within seven weeks after experimental exposure to *Bsal*, with *Bsal* infection loads increasing over time (Figure 2). In contrast, none of the five *C. asper* died, up until the end of the experiment at 16 weeks post inoculation. Only two animals developed detectable and fluctuating *Bsal* infections; one of these animals developed skin ulcerations. Both newts remained *Bsal* positive until the end of the experiment. Five of six *L. boscai* developed *Bsal* infection. One of six *L. boscai* died at 16 weeks post inoculation, whereas two animals had cleared infection at 20 weeks post inoculation and two animals remained *Bsal* positive for the entire duration of the experiment (20 weeks). None of the 15 *L. helveticus*, exposed to one of three *Bsal* isolates (including the *Bsal* type strain [11]), developed high level infections or clinical signs and none died. Only two *L. helveticus* exposed to the *Bsal* type strain (AMFP13/1) yielded a single borderline positive swab sample during the experiments; none exposed to the other isolates (AMFP14/1 and AMFP15/1) developed infections.

### 3.3. Inferring Bsal Susceptibility within and between Species

We found phylogeny to be related to a species’ susceptibility to *Bsal* infection, with Pagel’s λ equal to 0.639 (±0.001). This relationship was significant (*p* = 0.021) across 1000 replicates. This suggests that a species’ lab-determined susceptibility to *Bsal* infection is a reliable indicator of its closest relatives’ risk. We thus inferred susceptibility for species for which substantial empirical evidence is lacking (*L. maltzani* and *T. pygmaeus*) as intermediate and high, respectively) and we considered different lineages within a given species as equally susceptible. We therefore extrapolated the data from the lab trials, derived from a single source population, to all lineages of that given species.

### 3.4. Priority Areas and Lineages for Conserving Iberian Urodele Diversity

**Figure 3:**
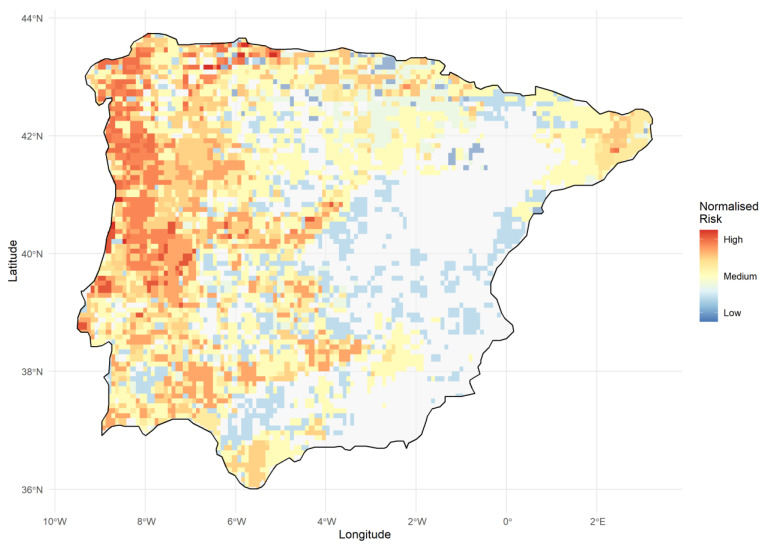
Spatial prediction of risk to Iberian urodeles from *Bsal* introduction. Each cell (10 km × 10 km) represents the normalized risk to biodiversity loss, given both species richness and the predicted susceptibility of inhabitants to *Bsal* infection. Darker red cells, as in the northwest/west regions of the peninsula, indicate areas likely to be most at risk should *Bsal* be introduced. Transparent gray cells are those with no salamander inhabitants.

Combining data from lab trials, mortality events in captivity, outbreaks in nature and phylogenetic modeling resulted in an estimated high susceptibility for five native Iberian urodele, intermediate susceptibility for four species and low susceptibility for one species (Table S2). One species (*C. asper*) was considered low-to-intermediately susceptible, given the development of intermittent high-level infections, accompanied by clinical lesions in only one animal. Two invasive species, *Triturus anatolicus* and *Ommatotriton nesterovi/ophryticus*, were classified as low and high, respectively. The predicted hotspots for urodele biodiversity loss given the introduction of *Bsal* are depicted in Figure 3.

We identified six conservation units at highest risk (Table 1): four small range and susceptible subspecies of the fire salamander (*S. salamandra*), the nominate subspecies of the golden striped salamander (*C. lusitanica lusitanica*) and the Montseny brook newt (*C. arnoldi*).

## 4. Discussion

Based on our results, *Bsal* currently appears to be restricted to a single previously described site in northeastern Spain, where mortality in *T. marmoratus* and *S. s. hispanica* was observed [12]. Outside of this area, none of our qPCR samples returned *Bsal*-positive results and no disease outbreaks were reported. In our assessment of species vulnerability, we demonstrate or infer that the majority of Iberian salamanders are susceptible to *Bsal* infection, suggesting a high likelihood of mass mortality events in most Iberian urodele species if *Bsal* becomes widespread. In the remainder of this paper, we discuss our results in the context of other recent *Bsal* reports from the Iberian Peninsula and provide recommendations to minimize disease outbreak risk and impacts.

### 4.1. Distribution of Bsal in the Iberian Peninsula

Our results contrast with two recent reports of positive qPCR *Bsal* results from northern Spain, not linked to obvious disease or mortality. The first report [14] mentions five positive skin swabs in palmate newts (*Lissotriton helveticus*). This study was followed by a second study sampling water bodies for eDNA, which yielded a high proportion of qPCR positive samples [13]. Despite our intensive sampling effort, we did not succeed in confirming these results in this region. Two exact localities were resampled and, besides a lack of *Bsal* detection, these sites hosted important and healthy populations of highly susceptible newts (*Triturus marmoratus*). Absence of disease outbreaks and low susceptibility of palmate newts ([11]; this study) raises doubts whether the positive PCRs results reported in [13,14] reflect the true presence of *Bsal*. False positive results have been reported for this diagnostic test [61,62,63] and may have far-reaching implications for conservation, since these may lead to the false conclusion that *Bsal* is apathogenic and widespread in wild urodele populations. Such conclusions are likely to affect the allocation of resources to mitigate the impact of this disease. For this reason, the OIE guidance explicitly specifies confirmation is needed using an independent diagnostic test (e.g., histopathology, culture) for a definitive *Bsal* diagnosis in a novel site or host species. The results of the current study support the validity of this requirement [26] and we urge caution interpreting conclusions based on qPCR-only results in novel sites or host species; such results should be treated as “*Bsal* suspect” until cross-validation has been conducted.

### 4.2. Susceptibility of Iberian Urodeles

By using all available lines of evidence, we predict the susceptibility of Iberian urodeles to *Bsal*. When no direct information was available, given we showed a strong phylogenetic signal for susceptibility, we used phylogenetic relatedness to infer susceptibility between different lineages of the same species and between species. We suggest considering different lineages of the same species equally susceptible given similar environmental conditions, which is further corroborated by evidence from *Bsal* outbreaks in captivity. During such outbreaks in collections of fire salamanders (*Salamandra salamandra*), mortality has been observed in most known subspecies, including several ones identified here as conservation units at high risk (*S. s. bernardezi, S. s. almanzoris, S. s. longirostris* [64,65]). For species for which no data (lab trials, outbreaks in nature or captivity) are available, we inferred susceptibility from its nearest relative. Of 11 Iberian species, we thus consider 10 susceptible to infection, five of which are predicted to be highly susceptible, suggesting significant population declines and the potential of population extirpation as witnessed in *Salamandra salamandra* in northern Europe. The likelihood of *Bsal* invasion is supported by predicted ecological niche overlap of *Bsal* with at least seven Iberian urodele species [24]. Since macroclimatic modeling obscures local host ecology and associated microclimatic conditions, *Bsal* may infect species that occur in cool and humid microhabitats within Mediterranean landscapes outside the pathogen’s predicted macroscale range [17,66]. Thus, we consider that all Iberian urodele populations could be at risk of infection by *Bsal* (i.e., broadscale climate refugia are unlikely to be present). However, this does not preclude the potential for local-scale microrefugia. Refugia would require environmental and body temperatures of the entire host community to exceed those of the thermal tolerance of *Bsal* long enough to kill the fungus entirely [17,67]. In practice, given the current understanding of the *Bsal* climate niche [17,66], a complete refugium would mean that the entire suitable host community and the infected environment maintain temperatures of 25 °C or more for at least 10 days. While lower temperatures that fail to eradicate the fungus may still have an impact by temporarily tempering virulence and slowing down fungal growth [15,16,67], such conditions could extend the infectious period of an infected host, facilitating fungal survival in the host population with the potential of disease flareups once temperatures drop.

### 4.3. Conservation Units for Iberian Urodeles

We define conservation units at highest risk of *Bsal-*driven extinction as evolutionary significant units of susceptible species, which occupy a range of less than 10,000 km^2^. While this threshold is arbitrary, it considers the relatively slow, but consistent natural spread of *Bsal* [68], as has been observed in Germany [69,70,71,72,73,74]. While the European action plan to mitigate *Bsal* infections [75] provides a Europe-wide risk assessment, we here used a more detailed geographic and genetic scale to develop region specific conservation strategies. Since the Iberian Peninsula has served as a regional glacial refuge during the Pleistocene, with multiple microhabitat refuges within it [76], this spatial complexity has contributed to the region being characterized as a European hotspot of diversity in the Salamandridae (e.g., [57]). The following six conservation units reflect this diversity at small scales, with representation of four subspecies of *Salamandra salamandra*, a range restricted subspecies of the monotypic *Chioglossa lusitanica* and the very small range *Calotriton arnoldi*. We propose Pleistocene delineation of these conservation units as a relevant and manageable, yet still relatively coarse scale. A more detailed scale could take into account more recent population structuring that has been demonstrated in a number of Iberian urodeles (Table 1) and may be preferred where resources are available.

Our proposed conservation units are based on current information, but new information could alter the list of priority lineages. For a few lineages, detailed data regarding genetic diversity and ranges occupied are currently missing, hampering proper delineation of conservation units. Current evidence suggests the existence of multiple evolutionary significant units for at least *T. marmoratus, T. pygmaeus, S. salamandra gallaica* and possibly *S. s. bejarae*. The acute threat of *Bsal* stresses the importance of in depth amphibian population assessments of genetic diversity and structure to maximize opportunities of persistence of salamander diversity.

### 4.4. Conservation Actions and Recommendations

The 2018 Bsal outbreak in the Iberian Peninsula was associated with mortality in at least two salamander lineages (*T. marmoratus*, *S. s. hispanica* [12]). The proximity of this outbreak to the distribution of Europe’s most threatened newt, the Montseny brook newt, alerted Catalan authorities who took swift action in collaboration with scientists and managers. A combination of host removal, fencing off the infected site, disinfection and setting a perimeter for disease surveillance contained the outbreak. Since mitigation success strongly depends on a rapid response, early detection of Bsal outbreaks is key [77,78]. Although active disease surveillance and population monitoring of susceptible populations across the Iberian Peninsula would be ideal, associated costs would be prohibitive. We therefore propose a more pragmatic, dual approach, combining passive reporting and fully comprehensive surveillance and population monitoring, coupled to the possibility of rapid disease diagnosis and mitigative actions [79]. Passive reporting would consist of centralized communication of urodele disease and mortality events across the Iberian Peninsula to a hotline by all stakeholders (including the general public, forest workers and scientists). Resources for active disease surveillance and population monitoring could be reserved for those areas where Bsal is predicted to cause the most pronounced biodiversity losses and/or highest risk of extinction of important conservation units (Figure 3).

The response required if *Bsal* is detected depends on the management objective. An ideal goal could be to maximize the probability of longtime persistence of all conservation units. Since *Bsal* containment, but not necessarily eradication, seems currently feasible [12], one focus could be on minimizing opportunities for further *Bsal* spread, even if this means large-scale host removal from the affected population to protect neighboring populations. Host removal requires either mass culling or setting up an ex situ program. While culling is commonplace for livestock, *Bsal* outbreaks involve wild and protected animals and such actions are prone to adverse public reactions. However, such options require consideration, as the long term ex situ management of large numbers of animals requires significant resources, with a high level of uncertainty of eventual rewilding success [12,80]. Maximizing the likelihood of success requires a clear decision context allowing fast decisions (including the necessary permits) and rapid initiation of drastic management actions [77]. We, therefore, call upon the Spanish and Portuguese authorities for preparedness through the development of a region-specific *Bsal* action plan that details the decision tree, clear attribution of responsibilities and the ability to quickly deploy resources and management actions when and where needed [78]. Such actions could consist of a combination of in situ and ex situ measures as detailed elsewhere [79] and should be maintained as long as the threat persists. Building capacity for ex situ conservation may be most useful for the six conservation units most vulnerable *Bsal-*driven extinction. The quick response to the Catalan outbreak and the presence of an ex situ conservation program for the Montseny brook newt (*Calotriton arnoldi* [81]) may serve as examples.

Given the expected loss of biodiversity and limited means of remedial action, preventing novel *Bsal* invasions is key. The risk of novel introductions could be reduced by promoting “clean trade” (absence of *Bsal* throughout the amphibian trade) by testing and treating captive amphibian collections throughout the commercial chain [12,65], improved biosecurity during field activities involving amphibians (including conservation actions like reintroductions or reinforcements, e.g., [82]) and controlling populations of invasive species. Invasive exotic urodele species (*Ommatotriton ophryticus/nesterovi, Triturus anatolicus*) in Spain have originated from released captive individuals [83] and are likely to have introduced *Bsal* in northern Spain in 2018 [12]. Anthropogenic movement of indigenous species (e.g., *I. alpestris* [40]; *C. lusitanica* [84]) equally carries the risk of pathogen spread. Since several of these species may develop chronic, high level infections, they may act as disease reservoirs that facilitate frequency-dependent *Bsal* transmission, which increases the probability of extirpation of highly susceptible species [15,85] and hence should be considered in risk assessments.

## Figures and Tables

**Table 1:** Iberian urodeles’ susceptibility to *Bsal* infection (1 = low, 2 = intermediate, 3 = high), an estimate of the number of 10 × 10 squares occupied by small range lineages (<100 squares) and their known genetic Pleistocene lineages as a proxy for conservation units. We highlight the existence of population genetic structure and other relevant information regarding the spatial genetic structure obtained from the literature (references) for each species/lineage. Shaded rows represent conservation units considered at highest risk.

(Sub-)Species	*Bsal* Risk	Number of 10 km × 10 km Squares	Pleistocene Lineages	Remark	Reference(s)
*Calotriton arnoldi*	3	2	1	Population genetic structure	[32,33]
*Calotriton asper*	2	>100	1	Population genetic structure	[34,35]
*Chioglossa l. longipes*	3	>100	1		[36,37,38]
*Chioglossa l. lusitanica*	3	<70	1		[36,37,38]
*Ichthyosaura alpestris cyreni*	2	>100	1	Population genetic structure	[39,40]
*Lissotriton boscai*	2	>100 for all clades	4	Population genetic structure	[41,42,43]
*Lissotriton maltzani*	2	>100	1		[41,42,44]
*Lissotriton helveticus*	1	>100 for all clades	4		[45]
*Pleurodeles waltl*	2	<30 for Algarve clade >100 for other clades	4	Small range population in Algarve somewhat differentiated but nuclear DNA shows admixed group covering a large area	[46,47,48]
*Salamandra salamandra almanzoris*	3	<30 for both clades	2	Population genetic structure	[49,50]
*Salamandra s. bejarae*	3	>100	2	Range of lineage not well known	[50] GV-A unpublished
*Salamandra s. bernardezi*	3	approx. 100 for the subspecies	2–7	High diversity in a small region; population genetic structure	[51,52,53,54] GV-A unpublished
*Salamandra s. crespoi*	3	<70	1		[55] GV-A unpublished
*Salamandra s. fastuosa*	3	>100	1		[53,56,57] GV-A unpublished
*Salamandra s. gallaica*	3	>100 for the subspecies	4	Range of lineage not well known; population genetic structure	[43,54,57,58] GV-A unpublished
*Salamandra s. hispanica*	3	>100	1		[57]
*Salamandra s. longirostris*	3	<100	3	Shallow lineages, considered one conservation unit; population genetic structure	[59]
*Salamandra s. morenica*	3	>100	1		[55] GV-A unpublished
*Triturus marmoratus*	3	>100 for the species	2	Range of lineage not well known	[60]
*Triturus pygmaeus*	3	>100 for the species	2	Range of lineage not well known	[60]

## Data Availability

All data are available in the manuscript and supplementary information.

## Supplementary Materials

The following are available online at https://www.mdpi.com/article/10.3390/jof7080644/s1, Table S1: Sampling of Spanish urodeles for the presence of *Bsal* between 2015 and 2021; Table S2: Susceptibility estimate of Iberian urodeles.
