# Supplementary material for: Batrachochytrium salamandrivorans Threat to the Iberian Urodele Hotspot
Source: J Fungi (Basel). 2021 Aug 7;7(8):644. doi: 10.3390/jof7080644 (PMC8400424; doi:10.3390/jof7080644)
Supplement: Supplementary file 1 [file jof-07-00644-s001.zip › jof-1304996-supplementary.pdf]

Journal of Fungi

Table S1: Sampling of Spanish urodeles for the presence of Bsal between 2015 and 2021. Car = *Calotriton arnoldi*, Ca = *Calotriton asper*, Cl = *Chioglossa lusitanica*, Ia = *Ichthyosaura alpestris*, Lb = *Lissotriton boscai*, Lh = *Lissotriton helveticus*, Pw = *Pleurodeles waltl*, Ss = *Salamandra salamandra*, Tm = *Triturus marmoratus*, Tp = *Triturus pygmaeus*.

Province_site	Year	Species	N	Bsal status	Sample type	Observations
Asturias_01	2015	Ia	5	Bsal negative	Toe-clip	
Asturias_02	2019	Ia	20	Bsal negative	Swab	
Asturias_03	2019	Lb	11	Bsal negative	Swab	
Asturias_03	2019	Cl	1	Bsal negative	Swab	
Asturias_04	2019	Ss	10	Bsal negative	Swab	
Asturias_05	2020	Lh	3	Bsal negative	Swab	
Asturias_05	2020	Tm	9	Bsal negative	Swab	
Asturias_06	2020	Lh	15	Bsal negative	Swab	
Asturias_07	2020	Lh	8	Bsal negative	Swab	
Asturias_07	2020	Tm	7	Bsal negative	Swab	
Asturias_08	2020	Lh	6	Bsal negative	Swab	
Asturias_08	2020	Tm	7	Bsal negative	Swab	
Asturias_09	2020	Lh	8	Bsal negative	Swab	
Asturias_09	2020	Ia	8	Bsal negative	Swab	
Asturias_09	2020	Tm	1	Bsal negative	Swab	
Asturias_10	2020	Ia	12	Bsal negative	Swab	
Asturias_11	2020	Lh	21	Bsal negative	Swab	
Asturias_11	2020	Tm	1	Bsal negative	Swab	
Asturias_12	2020	Lh	19	Bsal negative	Swab	
Asturias_13	2020	Ia	19	Bsal negative	Swab	
Asturias_13	2020	Ss	9	Bsal negative	Swab	
Asturias_14	2020	Lh	15	Bsal negative	Swab	
Asturias_15	2015	Ia	4	Bsal negative	Toe-clip	
Asturias_15	2020	Ia	11	Bsal negative	Swab	
Asturias_16	2020	Lh	10	Bsal negative	Swab	
Asturias_17	2020	Lh	2	Bsal negative	Swab	
Asturias_17	2020	Ia	7	Bsal negative	Swab	
Asturias_18	2021	Lh	1	Bsal negative	Swab	
Asturias_18	2021	Ss	20	Bsal negative	Swab	
Asturias_19	2021	Lh	2	Bsal negative	Swab	
Asturias_19	2021	Tm	2	Bsal negative	Swab	
Asturias_20	2021	Tm	12	Bsal negative	Swab	
Asturias_21	2021	Ss	20	Bsal negative	Swab	
Asturias_21	2021	Cl	20	Bsal negative	Swab	
Burgos_01	2017	Ss	7	Bsal negative	Toe-clip	Carcasses
Burgos_02	2020	Ss	2	Bsal negative	Toe-clip	Carcasses
Burgos_02	2020	Tm	2	Bsal negative	Toe-clip	Carcasses
Cantabria_01	2015	Ia	4	Bsal negative	Toe-clip	

Cantabria_02	2020	Ia	14	Bsal negative	Swab	
Catalunya_01	2020	Car	200	Bsal negative	Toe-clip	
Huelva_01	2018	Pw	46	Bsal negative	Swab	
Huelva_01	2018	Tp	77	Bsal negative	Swab	
Huesca_01	2020	Ca	26	Bsal negative	Swab	
Huesca_02	2020	Ca	7	Bsal negative	Swab	
Huesca_03	2020	Ca	17	Bsal negative	Swab	
Huesca_04	2020	Ca	10	Bsal negative	Swab	
León_01	2015	Ia	5	Bsal negative	Toe-clip	
León_02	2018	Ss	20	Bsal negative	Swab	
León_03	2019	Ia	18	Bsal negative	Tail-clip	
León_03	2019	Lh	1	Bsal negative	Tail-clip	
León_04	2020	Ss	19	Bsal negative	Swab	
León_05	2020	Lh	22	Bsal negative	Swab	
Lérida_01	2020	Ca	20	Bsal negative	Swab	
Lérida_02	2020	Ca	20	Bsal negative	Swab	
Madrid_01	2015	Ia	5	Bsal negative	Toe-clip	
Madrid_01	2018	Ss	8	Bsal negative	Swab	
Navarra_01	2015	Ia	4	Bsal negative	Swab	
Navarra_02	2020	Lh	5	Bsal negative	Toe-clip	Carcasses
Ourense_01	2020	Lb	20	Bsal negative	Swab	
Ourense_02	2021	Lb	15	Bsal negative	Swab	
Pontevedra_01	2020	Lb	4	Bsal negative	Swab	
Pontevedra_01	2020	Lh	8	Bsal negative	Swab	
Pontevedra_01	2020	Ss	18	Bsal negative	Swab	
Pontevedra_02	2020	Tm	10	Bsal negative	Swab	
Pontevedra_02	2020	Lb	3	Bsal negative	Swab	
Pontevedra_02	2020	Lh	8	Bsal negative	Swab	
Pontevedra_02	2020	Ss	2	Bsal negative	Swab	
Pontevedra_03	2020	Tm	16	Bsal negative	Swab	
Pontevedra_03	2020	Ss	29	Bsal negative	Swab	
Pontevedra_03	2020	Lb	2	Bsal negative	Swab	
Pontevedra_04	2020	Lb	20	Bsal negative	Swab	
Pontevedra_04	2020	Ss	1	Bsal negative	Swab	
Pontevedra_05	2020	Tm	1	Bsal negative	Swab	
Pontevedra_05	2020	Lb	1	Bsal negative	Swab	
Pontevedra_05	2020	Lh	4	Bsal negative	Swab	
Pontevedra_05	2020	Ss	3	Bsal negative	Swab	
Pontevedra_06	2020	Lb	4	Bsal negative	Swab	
Pontevedra_06	2020	Tm	18	Bsal negative	Swab	
Pontevedra_06	2020	Cl	4	Bsal negative	Swab	
Pontevedra_06	2021	Cl	7	Bsal negative	Swab	

Pontevedra_07	2020	Lb	22	Bsal negative	Swab	
Pontevedra_07	2021	Cl	1	Bsal negative	Swab	
Pontevedra_08	2020	Tm	1	Bsal negative	Swab	
Pontevedra_08	2020	Lh	23	Bsal negative	Swab	
Pontevedra_09	2020	Tm	14	Bsal negative	Swab	
Pontevedra_09	2020	Lb	5	Bsal negative	Swab	
Pontevedra_09	2020	Cl	1	Bsal negative	Swab	
Pontevedra_10	2020	Lb	16	Bsal negative	Swab	
Pontevedra_10	2020	Tm	6	Bsal negative	Swab	
Pontevedra_11	2020	Tm	18	Bsal negative	Swab	
Pontevedra_12	2020	Lb	8	Bsal negative	Swab	
Pontevedra_13	2020	Lb	20	Bsal negative	Swab	
Pontevedra_14	2020	Tm	6	Bsal negative	Swab	
Pontevedra_14	2020	Lb	17	Bsal negative	Swab	
Pontevedra_15	2020	Tm	2	Bsal negative	Swab	
Pontevedra_15	2020	Lh	3	Bsal negative	Swab	
Pontevedra_16	2020	Cl	1	Bsal negative	Swab	
Pontevedra_17	2021	Tm	6	Bsal negative	Swab	
Pontevedra_17	2021	Lb	19	Bsal negative	Swab	
Pontevedra_18	2021	Tm	7	Bsal negative	Swab	
Pontevedra_18	2021	Lb	14	Bsal negative	Swab	
Pontevedra_19	2021	Lh	53	Bsal negative	Swab	
Pontevedra_19	2021	Lb	1	Bsal negative	Swab	
Pontevedra_20	2021	Cl	2	Bsal negative	Swab	
Salamanca_01	2018	Pw	2	Bsal negative	Toe-clip	
Salamanca_01	2018	Lb	2	Bsal negative	Toe-clip	
Salamanca_01	2018	Tm	2	Bsal negative	Toe-clip	
Soria_01	2021	Lh	2	Bsal negative	Toe-clip	Carcasses
Soria_01	2021	Tm	3	Bsal negative	Toe-clip	Carcasses
Zamora_01	2018	Pw	32	Bsal negative	Toe-clip	
Zamora_01	2018	Tm	3	Bsal negative	Toe-clip	

Table S2. Susceptibility estimate of Iberian urodeles. Estimates are based on results from lab trials and mortality events in nature or captivity when available. *indicates a high level of uncertainty, with susceptibility mainly inferred from that of its sister species and (for *T. pygmaeus*) extensive mortality during a Bsal outbreak in captivity. **indicates an invasive species

Species	Death rate after experimental infection (infected)	Inoculum	Bsal isolate	Mortality in nature / captivity	Susceptibility	Reference
Calotriton arnoldi	5/5	10^3	AMFP18/2		high	Martel et al., 2020
Calotriton asper	0/5 (0/5)	10^3	AMFP13/1		Low-intermediate	This study
	0/5 (2/5)	10^3	AMFP13/1			
Chioglossa lusitanica	6/6	10^3	AMFP13/1		high	This study
Ichthyosaura alpestris	5/5	10^4	AMFP13/1	Nature and captivity	intermediate	Martel et al., 2014; Fitzpatrick et al., Schmeller et al., 2020
	1/5 (2/5)	10	AMFP13/1			Stegen et al., 2017
	1/5 (5/5)	10^2	AMFP13/1			Stegen et al., 2017
	2/5 (5/5)	10^3	AMFP13/1			Stegen et al., 2017
	5/5	10^4	AMFP13/1			Stegen et al., 2017
Lissotriton boscai	1/5 (4/5)	10^3	AMFP13/1	captivity	intermediate	This study, Fitzpatrick et al.
Lissotriton helveticus	0/5 (0/5)	10^4	AMFP13/1		low	This study
	0/5 (0/5)	10^4	AMFP14/1			This study
	0/5 (0/5)	10^4	AMFP14/2			This study
	0/5 (2/5)	10^4	AMFP15/1			This study
	0/3 (3/3)	10^4	AMFP13/1			Martel et al., 2014
Lissotriton maltzani	NA				Intermediate*	

*Ommatotriton nesterovi/ophryticus***	NA			captivity	high	Fitzpatrick et al., 2018
Pleurodeles waltl	3/8 (8/8)	10^3	AMFP13/1		intermediate	Greener et al., 2020
	5/5	10^4	AMFP13/1			Martel et al., 2014
Salamandra salamandra	6/6	10^3	AMFP13/1	Nature and captivity	High	This study, Martel et al., 2013; Fitzpatrick et al., 2018; Sabino Pinto et al. 2015
	9/9	10^3	AMFP13/1			Greener et al., 2020
	9/9	10^3	AMFP18/2			Martel et al., 2020
	40/40	10^2 (20); 10^4 (20)	AMFP13/1 (10); AMFP14/1 (10); AMFP14/2 (10); AMFP15/1 (10)			Stegen et al., 2017
	5/5	10^4	AMFP13/1			Martel et al., 2014
*Triturus anatolicus***	0/5 (2/5)	10^3	AMFP18/2		low	Martel et al., 2020
Triturus marmoratus	7/7	10^3	AMFP13/1	Nature and captivity	high	Greener et al., 2020; Fitzpatrick et al. 2018
	7/7	10^3	AMFP18/2			Martel et al., 2020
Triturus pygmaeus	NA			Captivity	High*	Fitzpatrick et al., 2018
